# Behind the Ink: Unmasking Asymptomatic Micro-perforations Following Endoscopic Tattooing

**DOI:** 10.7759/cureus.81995

**Published:** 2025-04-10

**Authors:** Woo Suk Kim, Tasur Seen, Joel Baum, Aaron Walfish

**Affiliations:** 1 Internal Medicine, Icahn School of Medicine at Mount Sinai, Queens, USA; 2 Gastroenterology, Icahn School of Medicine at Mount Sinai, Queens, USA

**Keywords:** endoscopic tattooing, gastroenterology and endoscopy, iatrogenic complication, incidental radiological finding, micro-perforation

## Abstract

Endoscopic tattooing is a valuable tool for identifying gastrointestinal lesions following initial discovery during endoscopy. Although generally considered safe, with minimal complications, routine post-procedure imaging is not practiced, and the detection of micro-perforation after tattooing is even rarer. We present a case of a 49-year-old male who underwent a colonoscopy for a workup of anemia, revealing a malignant-appearing mass in the ascending colon. The lesion was tattooed for further assessment. A follow-up CT scan for evaluation of metastatic disease incidentally demonstrated acute inflammatory changes and trace gas, consistent with micro-perforation. The patient was asymptomatic and hemodynamically stable on supportive treatment, with subsequent successful recovery. Therefore, close observation without additional interventions could be considered in patients with incidental micro-perforations and no symptoms. A large-scale study is needed to further demonstrate the correlation between endoscopic tattooing and micro-perforations to avoid unnecessary extensive evaluation.

## Introduction

Endoscopic tattooing is an accepted technique for precise localization of gastrointestinal lesions for further endoscopic or surgical intervention. It is considered a safe procedure, as the only notable adverse effect is local inflammation [1]. However, it is unclear whether tattooing can cause asymptomatic micro-perforations, as imaging is not routinely performed post-endoscopically.

Gastrointestinal micro-perforations are most frequently associated with iatrogenic instrumentation, such as endoscopic submucosal dissection, or complications of infectious processes, including diverticulitis and appendicitis [2,3]. They can lead to peritonitis from the spillage of intestinal contents into the abdomen and are treated with antibiotics, with or without emergent surgery [4]. 

Due to the potential mortality of peritonitis and high recovery rates with early intervention, the necessity of intensive evaluation and treatment of incidental micro-perforations should be explored. Although micro-perforations have the potential to develop into serious complications, close observation could be considered as an alternative management approach. We present a case of asymptomatic micro-perforation at the site of endoscopic tattooing to demonstrate that extensive workup may not always be necessary. 

## Case presentation

**Figure 1:**
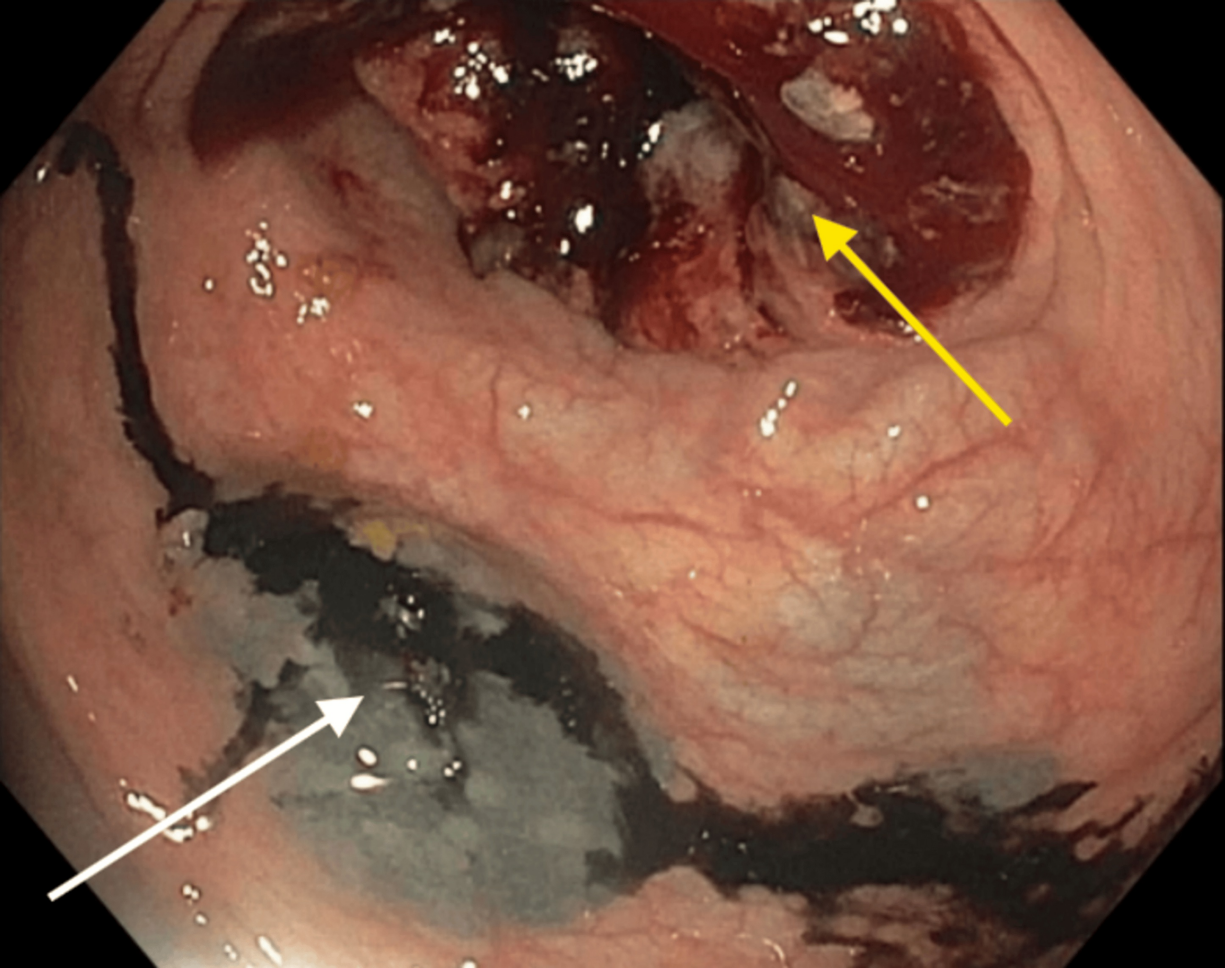
Endoscopic view of a circumferential, non-obstructing, malignant-appearing, ulcerated lesion in the proximal ascending colon (yellow arrow); non-India ink permanent marker proximal to the lesion for subsequent evaluation and intervention (white arrow).

A 49-year-old male with a history of iron deficiency anemia and alcohol dependence was hospitalized after routine blood work showed a hemoglobin count of 6.5 g/dL (reference range: 14.0-18.0 g/dL). He reported fatigue and dark stool for the past few years after starting oral iron supplements. Hemolysis and thyroid evaluations were normal. A colonoscopy for anemia workup revealed a circumferential, non-obstructing, malignant-appearing, ulcerated lesion in the proximal ascending colon. Biopsies were taken with cold forceps, and the site was tattooed with a non-India ink permanent marker using a standardized technique (Figure 1).

**Figure 2:**
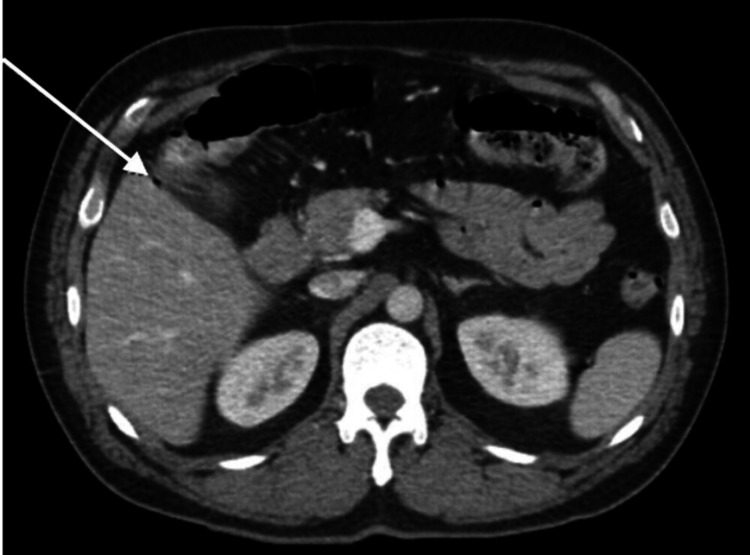
Axial view of the computed tomography scan of the abdomen, showing an intra-peritoneal air focus adjacent to bowel thickening, indicative of a micro-perforation at the site of endoscopic tattooing (arrow).

A CT of the abdomen was completed immediately post-endoscopy to assess for metastatic disease, which showed no metastatic lesions, but bowel thickening in the area of the ascending colon highlighted by the tattoo. The imaging was also noteworthy for acute inflammatory changes and trace gas adjacent to the bowel thickening within the highlighted region, consistent with a micro-perforation due to the tattoo (Figure 2). 

The patient remained asymptomatic, afebrile, and hemodynamically stable throughout the rest of the hospital course. Pathology was confirmatory of moderately differentiated invasive adenocarcinoma with invasion into the peri-colonic tissue. The patient subsequently underwent a right hemicolectomy a week later and was discharged without incident.

## Discussion

Radiologic imaging is not routine after endoscopic tattooing. However, if CT imaging is performed in an asymptomatic patient and a micro-perforation is incidentally discovered, close clinical monitoring is essential for early detection of symptoms and appropriate management. Unrecognized or untreated micro-perforations can lead to peritonitis, which could potentially increase mortality in patients who already have comorbid conditions, such as malignancies and infections [5].

A meta-analysis previously identified several complications of endoscopic tattooing, including infected hematoma or abscess formation, inflammatory pseudotumor, idiopathic inflammatory bowel disease, post-operative adhesions, and tumor inoculation. However, the study did not identify micro-perforation as a possible adverse effect [1]. The micro-perforation was not attributed to the biopsy, as prior prospective studies have not found perforation to be a complication of cold forceps biopsies [6]. This incidental discovery is similar to the established finding of asymptomatic pneumoperitoneum found on routine imaging after percutaneous endoscopic gastrostomy tube insertion, which does not necessitate any further intervention [7].

## Conclusions

Asymptomatic micro-perforation following endoscopic tattooing is a rare and likely under-recognized complication, as routine post-procedural imaging is not standard practice. Although endoscopic tattooing remains a safe and valuable modality for lesion localization, this case recognizes the merit of careful clinical assessment when incidental radiological findings, such as micro-perforation, are encountered. Additional studies are warranted to define the true incidence of micro-perforation following endoscopic tattooing and to establish management guidelines.
